# Wearables and Internet of Things (IoT) Technologies for Fitness Assessment: A Systematic Review

**DOI:** 10.3390/s21165418

**Published:** 2021-08-11

**Authors:** João Passos, Sérgio Ivan Lopes, Filipe Manuel Clemente, Pedro Miguel Moreira, Markel Rico-González, Pedro Bezerra, Luís Paulo Rodrigues

**Affiliations:** 1 ADiT-LAB, Instituto Politécnico de Viana do Castelo, Rua Escola Industrial e Comercial Nun’Álvares, 4900-347 Viana do Castelo, Portugal; joao.passos@ipvc.pt (J.P.); pmoreira@estg.ipvc.pt (P.M.M.); 2 IT—Instituto de Telecomunicações, Campus Universitário de Santiago, 3810-193 Aveiro, Portugal; 3 Escola Superior Desporto e Lazer, Instituto Politécnico de Viana do Castelo, Rua Escola Industrial e Comercial de Nun’Álvares, 4900-347 Viana do Castelo, Portugal; filipe.clemente5@gmail.com (F.M.C.); pbezerra@esdl.ipvc.pt (P.B.); lprodrigues@esdl.ipvc.pt (L.P.R.); 4 Instituto de Telecomunicações, Delegação da Covilhã, 1049-001 Lisboa, Portugal; 5 Department of Physical Education and Sport, University of the Basque Country, UPV-EHU, Lasarte 71, 01007 Vitoria-Gasteiz, Spain; markeluniv@gmail.com; 6 Research Center in Sports Sciences Health Sciences and Human Development, CIDESD, 5000-801 Vila Real, Portugal

**Keywords:** wearables, smart wearables, IoT, IoT in sports, fitness assessment

## Abstract

Wearable and Internet of Things (IoT) technologies in sports open a new era in athlete’s training, not only for performance monitoring and evaluation but also for fitness assessment. These technologies rely on sensor systems that collect, process and transmit relevant data, such as biomarkers and/or other performance indicators that are crucial to evaluate the evolution of the athlete’s condition, and therefore potentiate their performance. This work aims to identify and summarize recent studies that have used wearables and IoT technologies and discuss its applicability for fitness assessment. A systematic review of electronic databases (WOS, CCC, DIIDW, KJD, MEDLINE, RSCI, SCIELO, IEEEXplore, PubMed, SPORTDiscus, Cochrane and Web of Science) was undertaken according to the Preferred Reporting Items for Systematic Reviews and Meta-Analyses (PRISMA) guidelines. From the 280 studies initially identified, 20 were fully examined in terms of hardware and software and their applicability for fitness assessment. Results have shown that wearable and IoT technologies have been used in sports not only for fitness assessment but also for monitoring the athlete’s internal and external workloads, employing physiological status monitoring and activity recognition and tracking techniques. However, the maturity level of such technologies is still low, particularly with the need for the acquisition of more—and more effective—biomarkers regarding the athlete’s internal workload, which limits its wider adoption by the sports community.

## 1. Introduction

The concept of Internet of Things (IoT) emerged back in 1990 when the first device, a toaster, was connected to the internet to enable its remote control [1]. If in those days internet connectivity was the novelty, 30 years later, the term IoT represents a huge ecosystem that is far beyond connectivity, including multiple technologies (communications, computation, control, interaction), within several application domains, such as health, automation, industry and agriculture, but also in sports, where several studies have already been conducted [2,3,4,5] and where innovation and technology have been pushing the entire sports industry [6].

Bringing the IoT into sports opens a new era in athlete’s training, not only for performance monitoring/assessment but also for fitness assessment [2]. Typically, this is achieved through the inclusion of IoT wearable technologies that rely on sensor systems to collect, process and communicate information, such as biomarkers and/or other relevant indicators, that can be used to estimate the athlete’s capacity and evaluate the evolution of its physical and health conditions and therefore potentiate its performance.

Specifically, the application of IoT solutions in sports and fitness has allowed simplifying data acquisition processes with the use of wearables that allow a faster and more efficient improvement in the athlete’s training. These devices are carried by athletes in the form of clothing or accessories and are designed to include sensors, a microprocessor and a communication unit that enables connectivity within a personal area network (PAN) where the smartphone plays a central role, not only for data storage and processing but also to operate as a gateway, empowering wearable devices with ubiquitous connectivity to the internet.

The use of biomarkers in sports and fitness allows the use of specific characteristics that are measured and used as an indicator of normal biological processes, pathogenic processes, or responses to a specific external exposure or intervention. There are several biomarker subtypes that can be defined as an identity, a biologic plausibility and its measurement method [7]. In this sense, IoT technologies make the collection, processing, communication and storage of these biomarkers easier, empowering the digital transformation in sports and fitness, and making digital biomarkers more objective, due to their real-time nature, real-world applicability and data availability [8]. Biomarkers are often collected by wearables and aggregated by smartphones, and given the recent advances of machine learning and Artificial Intelligence (AI), new avenues for knowledge extraction from biomarkers data arise, pushing research and technology towards a new era in sports and fitness assessment [8].

Fitness assessment and training load monitoring have become a popular topic of research in sports sciences [9,10,11]. These areas help the coaches to better understand the status of the player, as well as the functional adaptations over time [12,13]. While fitness assessment represents a moment in time (picture), the monitoring process occurs in a continuum over the period of intervention/exposure. Usually, fitness assessment in athletes covers the main physical abilities, namely, aerobic capacity (also known as cardiorespiratory fitness), anaerobic capacity and power, neuromuscular capacity (strength and power), speed and change-of-direction and mobility [14]. Additionally, anthropometric and postural assessments are also common in a complete battery of fitness assessments commonly performed in athletes [15].

Regarding athletes’ monitoring, normally, four main areas are covered [16]: (i) internal load, (ii) external load, (iii) well-being and (iv) readiness. Internal load represents the psychophysiological responses to a given external load, while external load represents the physical demands associated with a given stimulus provided by the coach [17]. Internal loads are typically monitored using oxygen uptake, blood lactate concentration, heart rate, or rate of perceived exertion [18]. The external loads are typically monitored using global navigation satellite systems, inertial measurement units (IMU) [19], or linear transducers that provide measures related to distances covered at different speed thresholds, accelerations/decelerations and changes-of-direction [20]. Well-being is typically monitored using subjective scales related to fatigue, stress, quality of sleep, delayed onset muscle soreness, or mood. However, sleep can also be measured using accelerometry [21]. Readiness is normally assessed using the heart rate variability, heart rate recovery, variations on neuromuscular tests (using force plates, or contact platforms), or variations in maximal efforts (such as cycling or sprinting) [22,23].

Current wearable and IoT technologies are used in sports for monitoring both the internal and external workload of athletes. However, there is still a need to obtain more information about the athlete’s internal workload, which is crucial to adjust training and increase the athlete’s performance. For example, the possibility to monitor physiological biomarkers, such as saliva or sweat, in a non-invasive and continuous manner, enables the possibility for optimal hydration adjustment, enhancing the overall athlete’s performance [24]. Furthermore, the use of such technologies opens new possibilities regarding activity recognition [25,26,27,28,29,30] and activity tracking [31,32,33,34] in sports.

Both processes, fitness assessment and athlete monitoring, can provide a great amount of data, pending the type of instruments used. However, the way that these data are connected and exported is relevant, namely to make the process of information extraction and report.

Thus, considering the importance of wearables and IoT technologies for fitness assessment, this systematic review presents the following main contributions: (i) identification and summarization of studies that have used wearables and IoT technologies for fitness assessment and (ii) discussion of the examined studies in terms of applicability of the used technologies for fitness assessment.

The remainder of this article is organized as follows: Section 2 presents the materials and methods used in this study. Section 3 presents the results obtained. Section 4 presents the results discussion, and lastly, in Section 5 the main conclusions are undertaken.

## 2. Materials and Methods

This systematic review was prepared according to the guidelines defined in PRISMA (Preferred Reporting Items for Systematic Reviews and Meta-analysis), cf. [35]. The adopted protocol has been registered on the International Platform of Registered Systematic Review and Meta-Analysis (https://inplasy.com/), accessed on 13 June 2021, with the number INPLASY202160041 and the DOI number 10.37766/inplasy2021.6.0041.

### 2.1. Eligibility Criteria

The search protocol was conducted independently by two authors (F.M.C. and M.R.-G.) to identify potentially relevant studies, which consisted of the evaluation of the title, abstract and reference list of each study. The inclusion and exclusion criteria can be found in Table 1. Moreover, the full versions of papers included in the study were revised in detail to identify—and consequently remove—the articles that do not meet the selection criteria. An additional search within the list of references of the included papers was conducted to retrieve additional relevant studies, and a final discussion was made in the cases of discrepancies regarding the selection process with a third author (S.I.L). Possible errata for the included articles has also been considered.

### 2.2. Information Sources and Search

Several electronic databases (WOS, CCC, DIIDW, KJD, MEDLINE, RSCI, SCIELO, IEEEXplore, PubMed, SPORTDiscus, Cochrane and Web of Science) have been searched for relevant publications prior to 9 March 2021, the day when all the searches were performed. Keywords and synonyms were entered in various combinations in the title, abstract, or keywords: (sport OR exercise OR “physical activity”) AND (“wireless body sensor network” OR WBSN OR smartwatch* OR watch OR clothing OR tracker* OR footwear OR wearable* OR “inertial measurement unit” OR IMU OR MEMS OR microelectromechanical OR accelerometer OR gyroscope OR barometer) AND (IoT OR “Internet of Things”) AND (performance OR movement* OR behavior* OR fitness OR cardio* OR aerobic* OR strength OR neuromuscular OR sprint* OR agility OR change-of-direction OR “heart rate” OR HR).

Further, the reference lists of the selected studies were manually screened to identify potentially eligible works not identified during the search in the electronic databases. Subsequently, an external expert was contacted to verify the final list of references included in this scoping review to identify possible relevant studies not detected by our search. Possible errata were searched for each included study.

### 2.3. Data Extraction

A spreadsheet was prepared for data extraction following the guidelines of Cochrane Consumers and Communication Review Group’s [36]. The spreadsheet was used to identify the accomplishment of inclusion or exclusion criteria and to support the selection of the articles. The process was made by two of the authors (F.M.C. and M.R.G.) independently. Following, both authors compared the results, and in case of any disagreement regarding the eligibility of a specific work, a discussion was undertaken and a final decision was made upon agreement.

### 2.4. Data Items

In the analysis performed on the selected articles, the following data items were extracted: type of study design, number of participants (N), age group (mean ± SD), sex (male, female, or both), experimental protocol and type of exercise; characteristics of the wearable device (sensors, actuators, microcontroller, processor, network topology), (iii) characteristics of the software tools (software, ML algorithms, IA mechanisms).

### 2.5. Methodological Assessment

The STROBE assessment was applied by two authors (F.M.C. and M.R.-G.) to evaluate the methodological bias of the eligible articles by following the adaptation of O´Reilly et al. [37]. Each of the included articles was scored for ten items, cf. [37]. The assessment was made independently, and in case of disagreement, a discussion was undertaken and a decision was made upon consensus among the authors. Following this, both authors compared the results obtained, and any disagreement regarding the scores was discussed and a decision was made according to agreement by consensus. Each study was rated qualitatively following the O´Reilly et al. methodology [37]: from 0 to 6 points, the study was considered at risk of bias (low quality), and, from 7 to 10 points, the study was considered as having a low risk of bias (high quality).

## 3. Results

This section is divided into four subsections that include the study identification and selection; the assessment of the methodological quality; the identification of the individual characteristics of the studies; and finally, the extraction of the final results of the individual studies.

### 3.1. Study Identification and Selection

**Figure 1:**
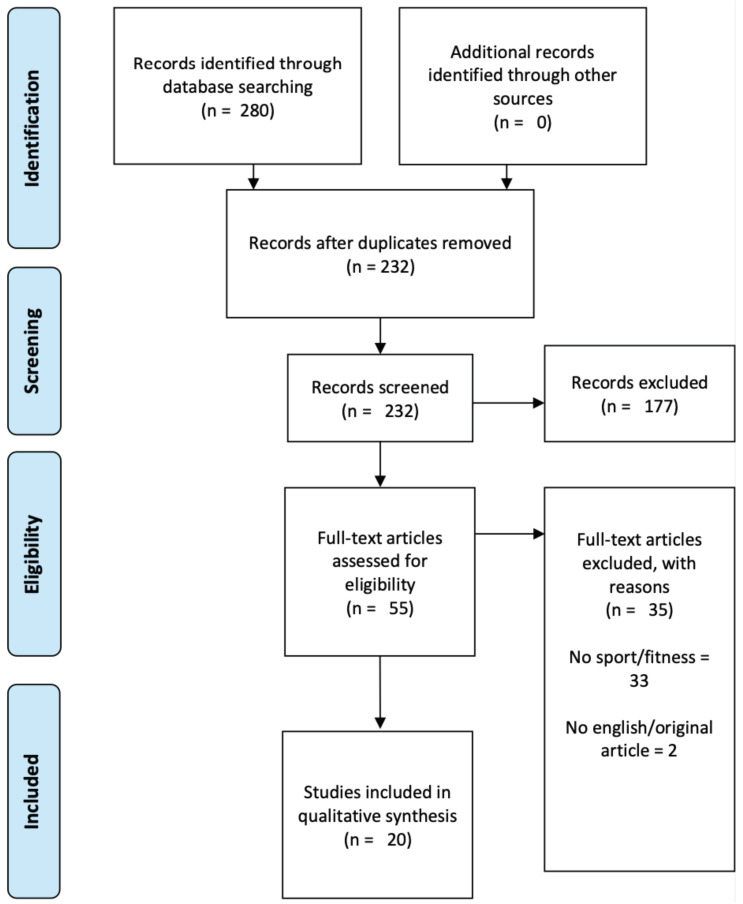
PRISMA Flow Diagram.

The database searching identified a total of 280 titles (IEEExplore = 132; Cochrane = 2; PubMed = 36; SPORTDiscus = 1; Web of Science = 61). These studies were then exported to a reference manager software (EndNoteTM X9, Clarivate Analytics, Philadelphia, PA, USA), and 48 duplicates were removed either automatically or manually. Following this, the remaining 232 articles were screened for their relevance based on their title and abstract, which resulted in the elimination of 177 additional studies. After the screening procedure, 55 articles were selected for in-depth reading and analysis. After reading the full texts, a further 35 studies that did not meet the eligibility criteria were excluded. The PRISMA Flow Diagram that represents the adopted search methodology is presented in Figure 1.

### 3.2. Methodological Quality

The methodological assessment revealed that seven (35%) articles had low overall quality, while 13 (65%) had high quality. The specific scores can be observed in Table 2.

### 3.3. Characteristics of the Individual Studies

After a review of the included studies, information was collected regarding the wearable/IoT device (sensors, processors, memory, etc.), the software(s) used, the machine learning algorithms and finally the fitness assessment method used in each study. Of the devices presented, the one with the most studies was the wristband, while the remaining studies covered wearables such as gloves, t-shirts, watches, waistband, chestband and calfband. Of the devices presented, the one with the most studies was the wristband, while the remaining studies covered wearables such as gloves, t-shirts, watches, waistband, chestband and calfband. Only four studies presented commercial devices. Of the studies that used Machine Learning algorithms, nine used Support Vector Machine, four used Convolutional Neural Network, another four used Decision Tree and another four used K-Nearest Neighbor and in some studies more than one algorithm was used. More details on the characteristics of the studies are presented in Table 3.

### 3.4. Results of Individual Studies

The results extracted from the studies, cf. Table 4, are based on the characteristics of wearable and/or machine learning algorithms. After the analysis, it was found that only three of the included studies were not conducted with the scope of the wearable but rather on the effectiveness of the ML algorithm.

## 4. Discussion

This systematic review aimed to identify and summarize studies that have examined the applicability of wearable and IoT devices for fitness assessment.

Overall, eleven distinct wearable/IoT devices types have been evaluated for fitness assessment. The examined studies were conducted using a glove [38,57], wristband [39,40,41,46,49,52,53], calf band [42], bicycle [44], waistband [45], chest band [46], smartwatch [47,54], smartphone attached to belt [48], T-shirt [50], upper torso strap [51] and bracelet [53]. Two studies did not report any results regarding the use of wearable/IoT devices for fitness assessment [44,47]. To discuss the technologies under analysis, we opted to evaluate the examined works based on the study characteristics presented in Table 3, taking into account relevant criteria for the implementation of the devices and their impact on the application side. The discussion will be based on the next four criteria:

A. Sensing: suitability of the used sensors for biomarkers acquisition;
B. Processing: computational capacity and its impact on the device’s autonomy;
C. Communications: communications protocols and their impact on the device’s autonomy and security and privacy;
D. Applicability: applicability of the wearable/IoT technology for fitness assessment.

Typically, wearable and IoT devices are carried by athletes in the form of clothing or other accessories designed to include sensors, a microprocessor and a communication unit that enables connectivity with a smartphone or a third-party service provider, and demand for small footprint, a powerful CPU for intermittent processing (i.e., quick data processing with fast return to a deep sleep state) with low-power consumption and low-power communications for ubiquitous interoperability. In such architectures, the smartphone plays a central role, not only for data storage and processing but also to operate as a gateway, empowering wearable devices with ubiquitous connectivity to the internet.

Regarding criterion A, the suitability of the used sensors for biomarkers acquisition, it is still important to develop integrated sensing technologies, namely with a focus on the miniaturization at the Integrated Circuit (IC) level, which includes the design of System on Chip (SoC) ICs for data acquisition, pre-processing and wireless communications [58,59]. Since wearables are physically attached to the athlete’s body, removing wires can be of great value regarding the applicability of such systems in a real-world scenario. Therefore, designing specific ICs that integrate sensor technologies (i.e., that include an analog frontend for sensor interfacing), a powerful CPU (for intermittent processing) and a low-power radio (for intermittent communications) is a direction that should be considered. To achieve increased reliability, sensor technologies need to be focused on the improvement of the signal-to-noise ratio and sensitivity, which may demand from IC manufacturers new possibilities such as new IC SoC packaging approaches that aim to pursuit more reliable and robust wearable devices, cf. [60,61].

Regarding criterion B, reducing the overall power consumption of wearable/IoT technologies is crucial to achieving higher maturity levels. However, only two of the examined works [38,42] evaluated the autonomy, which can be observed by the fact that the majority of the evaluated works are still at the prototype stage. Additionally, the convergence towards the design of Application Specific Integrated Circuits (ASICs) will help to reduce the overall power consumption at the same time that integration and miniaturization will pave the way to less invasive wearable and IoT devices in sports. Additionally, and as a result, the overall cost of such devices will be reduced, since large-scale production tends to reduce the overall production cost.

Regarding criterion C, the majority of the examined works use communication standards designed to operate in Local Area Networks (LAN) using Wi-Fi [47,48,49] and Zigbee [53]) protocols, or in a Personal Area Network (PAN) using the Bluetooth [41,46,53,54] and Bluetooth Low-Energy (BLE) [38,40,44,49,52]) communication protocols. Only one work uses a Wide Area Network (WAN), i.e., 4G mobile communications [56]. When looking at the impact of the communication protocols on the device’s autonomy, it is important to keep in mind that, from the examined protocols, only the Zigbee and the BLE protocols have been designed for low-power operation, meaning that all the works that rely on other communication protocols will have a reduced autonomy mainly biased by the adopted communication protocol. Another important factor that was not evaluated in this study was the communication delay, which is heavily dependent on the adopted communications technology used, due to the fact that none of the examined works addressed this issue. Regarding security, Wi-Fi uses 256-bit encryption, whereas Bluetooth and LE use only 128-bit encryption, which is the common level of security that standard applications do require. However, if a high level of security is required, Wi-Fi must be considered with Wireless Equivalent Privacy (WEP) and Wi-Fi Protected Access (WPA2-AES), which make the communications demonstrably safer [62].

Regarding criterion D, among the examined studies that have reported results for fitness assessment, two major categories have been identified—(1) Physiological Status Monitoring with five works [40,41,50,53,56] and (2) Activity Recognition/Tracking with eleven works [39,42,43,45,46,48,49,51,52,54,57]—which will be discussed separately in the following two subsections.

### 4.1. Wearables and IoT Technologies for Physiological Status Monitoring in Fitness Assessment

From the first category Physiological Status Monitoring, several wearable and IoT technologies (wristband [40,41,53]; T-shirt [50]; bracelet [53]) have been used along with distinct processing/machine learning approaches, cf. Table 4.

Three of the examined works [40,41,53] have used a wristband—all in prototype phase—for physiological status monitoring. More specifically, in [40], Brueck et al. present a prototype using a wristband with a calorimetric flow rate sensor that has been interfaced with a Raspberry Pi to send sweat rate information data to the cloud for athlete hydration monitoring. In [41], a prototype based on a calf band equipped with a motion sensor was used along with a machine learning fitness evaluation model oriented for teenager running monitoring. Xiao et al. in [53] use a wristband to monitor athletes’ health by acquiring their heart rate and transmitting it to a smartphone or computer for storage and further analysis.

The two remaining works that rely on the first category include a T-shirt [50] and a bracelet [53]. The former introduces a prototype based on a t-shirt that was embedded with sensors for heart rate monitoring along with acceleration. Data are acquired and transferred to Cloud services to be further classified by a machine learning model in order to get a prediction of an athlete’s heart rate. The latter presents a prototype that assists mountaineering guides in leading mountaineering teams by collecting information about those teams such as body temperature and heart rate. Through 4G, the information is uploaded to a Cloud network management platform to store data and enable location services.

The use of physiological status monitoring plays a determinant role in the individualization of the training process. One of the most common markers used to monitor and adjust the training intensity is the heart rate, and these new possibilities of using wristbands or t-shirts reduce the discomfort used by chest bands traditionally associated with heart rate monitors. Additionally, adding information about sweat rate may also indicate how the hydration should be replaced, aiming to adjust to the environment and consequences of exercise. The combination of these indicators with data processing using machine learning may allow faster identification of critical zones or target zones for training in respect to each participant, namely considering their history for similar conditions. This may, in the future, make it possible to design training conditions or even to determine the needs of dietary and hydration before and after exercise, making it adjusted to the participant. Possibly, in the future, the combination between heart rate and acceleration-based data will allow a better understanding of the dose–response relationship between external load (dose) and the consequence in internal load (response), which may vary from athlete to athlete considering the fitness baseline levels.

### 4.2. Wearables and IoT Technologies for Activity Recognition/Tracking in Fitness Assessment

Regarding the second category Activity Recognition/Tracking several wearable and IoT technologies (wristband [39,46,49,52]; calf band [42]; waistband [45]; chest band [46]; smartphone attached to belt [48]; upper torso strap [51]; glove [57], smartwatch [47,54]), have been in use along with distinct processing/machine learning approaches, cf. Table 4.

Seven of the examined works [39,42,45,46,49,52] have used wearable bands (wrist, calf, waist and chest) for activity recognition/tracking. In the first, Barricelli et al. [39] used a commercial FitBit charge HR for heart rate monitoring, step counting, physical activity monitoring and sleep detection. These data are then transmitted to the cloud to create a human Digital Twin (DT) that is continuously fed by the athlete’s fitness-related measurements. After collecting enough data, the DT predicts the athlete’s performance during training, and, depending on its performance, changes in the athlete’s behavior can be suggested. The second work [46], introduces a prototype developed to recognize exercises performed in a gym. This prototype consists of a set of two devices, one placed on the wrist and the other on the chest area. Both devices have an accelerometer, and in addition to this, the sensor placed on the chest also allows the reading of ECG signals. The data collected are preliminarily classified to distinguish aerobic exercises from free weight exercises. After this distinction, and for each of the classifications, the prototype will be able to count repetitions and series of free weight exercises, as well as recognize the aerobic activity performed or even sedentary activity. In the third work [49] a prototype based on a wristband was developed with inertial sensors for recognizing classic racket sports movements. The data collected by the wristband are sent to a smartphone via Bluetooth Low Energy, which relays them to a remote server in the Cloud for further analysis and querying. In the fourth work, [52] a prototype is proposed consisting of a wristband embedded with accelerometers and gyroscopes and a multilayer hybrid clustering model to achieve regular motion recognition of racket sports. The data collected by the wristband are sent to a smartphone via BLE for consulting or sharing with others. In the fifth work [42], Huang et al. present a prototype based on a calf band that was integrated with motion sensors to enable detection of physical activity by collecting the data to a computer and then classifying them using ML-based algorithms. In [45], the authors present a prototype that was incorporated with a three-axis accelerometer for collecting data regarding an individual’s movement. After storage, CNN is applied for extraction of the relevant features for recognition and characterization of the physical activity that is performed. Lastly, in [43] the authors present a prototype designed with tri-axial sensors for data acquisition. These data are later segmented for feature extraction, which is done in two different ways: the first is done by encoding an image, and the second is done manually. Both extractions are concatenated and classified using SVM.

The four remaining works that fit into the second category include a smartphone attached to belt [48], an upper torso strap [51], a glove [57] and a smartwatch [47,54]. In [48], Sun et al. present a prototype consisting of inertial sensors and a depth camera to recognize an athlete’s behavior on and off the field. The prototype consists of a ribbon where a cell phone is attached for data collection. The data from both sensors are first segmented to be later classified using ML algorithms. In the second work, [51], a prototype based on a chest band with accelerometers was developed for injury recognition and prevention in athletes. With previously entered data, this device is able to evaluate the athlete’s posture when performing exercises in order to predict the risk of injury. All the data are collected by a smartphone that later sends them to a server in the Cloud. The third work [57], consists of a glove prototype that has embedded inertial sensors for activity recognition and non-standard behavior detection with data being presented to a mobile application for quality assessment and analysis. Lastly, the work [54] presents a solution for human motion recognition during Ping Pong practice using a commercial smartwatch. This device allows the collection of data such as acceleration, angular velocity and magnetic field strength that are sent to a smartphone and a computer for further classification with ML algorithms.

Reducing the devices to differentiate the modality of exercise and quantify the external load and impact on the athlete is one of the main priorities in the future for sports sciences applications. The introduction of IMU allows minimizing the use of mechanical instruments that are typically less friendly and portable. As mentioned above, the use of belts, straps, gloves, or smartwatches is a step forward for making data quantification easier and more friendly. However, the benefit of using these devices is, precisely, that they integrate the capacity of establishing the connection with cloud solutions and data processing. In fact, the adjustment of training based on this immediate process may be a great solution for recreational athletes or even for professionals. For example, the velocity-based training allows, currently, to determine the number of repetitions in a set with the control of maximum loss of acceleration. This ensures higher performance, mainly for cases of weight lifting exercises made at the maximum intention. The same for throwing exercises. The capacity of the devices to detect these changes and make additional treatment to determine the appropriate load and repetitions for an athlete may be a step forward, namely using machine learning. Additionally, in the future, the capacity to recommend the weekly frequencies, the most appropriate exercises, or the automatic adjustment of load may be a step forward that helps anyone that works without a strength and conditioning coach.

### 4.3. Study Limitations, Future Research and Practical Implications

The majority of the works examined in this systematic review have been conducted keeping in mind the application of Physiological Status Monitoring and Activity Recognition/Tracking to fitness assessment. The focus of this systematic review is to evaluate the applicability of wearable and IoT technologies for such applications in terms of their hardware, software and processing mechanisms, such as machine learning or other relevant tools. However, the current use of wearable and IoT technologies in sports to monitor athletes’ internal and external workload is still in development. The need to obtain more—and better quality—information about the athlete’s internal workload, is still unmet by the research community [24], which can be justified by the fact that most of the studies examined in this article are still prototypes (16 out of 19), revealing that the maturity of such technologies is still low. Moreover, the rise of machine learning in sports can improve considerably the utility of wearable and IoT technologies and help to pave the way for the next mile into predictive fitness analytics [63,64,65,66]. ML-based techniques, such as Regression Analysis (e.g., Decision Trees [54], Random Forest [54]), Classifier Methods (e.g., SVMs [39,42,48] Nearest Neighbor [41,54]) and Clustering Methods (e.g., K-means [52], Neural Networks [46,54] and Hierarchical Clustering [52]), are examples of mature technologies that can be applied into predictive fitness analysis modeling and learning.

Additionally, new advances in flexible electronics and IC fabrication are transforming the development of wearables and IoT devices. However, there are still limitations associated with measuring several biomarkers that are challenged by the limitations presented not only at the physical sensor implementation level but also at the system’s edge computing level, namely with the need for lightweight machine learning implementations for effective data analytics at the edge. Another important issue that needs to be addressed is related to the autonomy of such devices. For example, reducing the overall power consumption of the device is crucial for achieving a higher maturity level and surpass the initial prototype stage.

## 5. Conclusions

Wearable and IoT technologies have been used in sports to monitor both the internal and external workloads of athletes. However, the collection of more biomarkers regarding the athlete’s internal workload is crucial to effectively adjust training and increase the athlete’s performance. Furthermore, another important observation of this study is that the maturity of such technologies is still low, which ends up conditioning its adoption by the sports community in a wide way.

On the other hand, physiological status monitoring and activity recognition/tracking open up new possibilities regarding fitness assessment; notably, with the recent advances in machine learning in sports, predictive fitness analytics is becoming a consistent trend by enabling the use of predictive models to determine appropriate training and in-game strategies.

This systematic review allowed us show that internal and external load have been collected and analyzed separately. Future advances should add machine learning techniques to determine relationships between those variables and determine the optimum and individualized training targets for recreational and professional athletes, helping them to monitor and adjust the training process to the individual conditions and environmental factors.

## Figures and Tables

**Table 1:** Eligibility criteria.

Inclusion Criteria	Exclusion Criteria
Applications of wearable and IoT in fitness assessment (i.e., assessment of cardiorespiratory level, neuromuscular status, balance, sprint and change-of-direction, body mass or body composition) and health monitoring in athletes or sports (e.g., hearth rate, sleep quality).	Applications of wearables and IoT in other human activities not related to fitness assessment or health monitoring in athletes (e.g., healthcare monitoring, well-being monitoring not related with sports, clinical populations, medical devices.
Only original and full-text studies written in English.	Written in other language than English. Other articles types than original (e.g., reviews, letters to editors, trials registrations, proposals for protocols, editorials, book chapters and conference abstracts).
	The studies must specify the hardware and software of the wearable/IoT device and include the adopted fitness assessment method.

**Table 2:** Methodological assessment of the included studies.

References	1	2	3	4	5	6	7	8	9	10	Quality
Akpa et al. [38]	1	1	1	1	1	1	1	1	1	1	High
Barricelli et al. [39]	1	1	0	1	1	1	1	1	1	0	High
Bruek et al. [40]	1	0	0	1	1	0	1	1	1	1	High
Guo et al. [41]	1	0	0	1	1	0	1	0	0	1	Low
Huang et al. [42]	1	0	0	1	1	0	1	1	1	0	Low
Huynh-The et al. [43]	1	0	0	1	1	0	1	0	1	1	Low
Municio et al. [44]	1	0	0	1	1	1	1	0	0	1	Low
Muñoz-Organero et al. [45]	1	0	1	1	1	1	1	0	1	1	High
Qi et al. [46]	1	0	1	1	1	1	1	1	1	0	High
Roslan & Ahmad [47]	1	0	0	1	1	0	1	1	1	0	Low
Sun et al. [48]	1	0	1	1	1	0	1	0	1	1	High
Wang et al. [49]	1	0	1	1	1	1	1	0	0	1	High
Wang & Gao [50]	1	0	0	1	1	1	1	0	0	0	Low
Wilkerson et al. [51]	1	0	1	1	1	1	1	1	1	0	High
Xia et al. [52]	1	0	1	1	1	1	1	1	1	1	High
Xiao et al. [53]	1	0	0	1	1	0	1	1	1	1	High
Zhang et al. [54]	1	0	1	1	1	1	1	1	1	1	High
Zhang et al. [55]	1	0	0	1	1	0	1	1	1	0	Low
Zhao et al. [56]	1	1	1	1	1	1	1	0	1	1	High
Zou et al. [57]	1	0	1	1	1	0	1	1	0	1	High

Note: Provide in the abstract an informative and balanced summary of what was done and what was found (item 1). Give state-specific objectives, including any prespecified hypotheses (item 2). Give eligibility criteria and the sources and methods of selection of participants (item 3). For each variable of interest, give sources of data and details of methods of assessment (measurement). Describe comparability of assessment methods if there is more than one group (item 4). Explain how quantitative variables were handled in the analyses. If applicable, describe which groupings were chosen and why (item 5). Give characteristics of study participants (item 6). Summarize key results with reference to study objectives (item 7). Discuss limitations of the study, considering sources of potential bias or imprecision. Discuss both direction and magnitude of any potential bias (item 8). Give a cautious overall interpretation of results considering objectives, limitations, multiplicity of analyses, results from similar studies and other relevant evidence (item 9). Give the source of funding and the role of the funders for the present study and, if applicable, for the original study on which the present article is based (item 10).

**Table 3:** Study Characteristics.

			Wearable/IoT Hardware							Software Tools (Edge/Cloud Computing)		Fitness Assessment		
Study	Device Type	Commercial/ Prototype	Sensors/ Atuators	Processor/ Memory/DevBoard	Communication Protocols	Network Topology	Autonomy	Online/ Offline	Biomarkers	Analytics/ML/AI	Software	Type of Exercise	Experimental Protocol	Population N/Sex/Age
Akpa et al. (2019) [38]	Glove	Prototype	Force-sensitive resistor (FSR)	MCU Adafruit Feather: Processor: Atmega32u4 Memory: 32 kB Flash 2 kB RAM	BLE	Star	6 h	Offline	Hand Pressure Distribution	Algorithms: Decision tree SVM k-NN	UI for data display	Bench dips Climber Dumbbell curl Knee-pull-in Knee-twist-in Plank leg raise Pilate dips Push-up Side-to-side lunge Wall push-up	1 h workout 10 exercises 3 sets p/ exercise 1–2 min. rest	N = 10 S: both A: 25.9 ± 3.21
Barricelli et al. (2020) [39]	Wristband FitBit Charge HR	Commercial	n.d.	n.d.	n.d.	P2P	n.d.	Offline	Heart rate number of steps per day physical activity sleep	ML classifiers: svm-based knn based	n.d.	Soccer, strength and conditioning	3-day measurements w/1 training session at the 3rd day	N = 10 S: male A: 19
Brueck et al. (2018) [40]	Wristband	Prototype	Calorimetric flow rate sensor w/a Macroduct sweat collector	Processor: 32-bit ARM Cortex-M0 Memory: 128 kB Flash 24 kB RAM DevBoard: Raspberry Pi	BLE	Star	n.d.	Both	Sweat	n.d.	ThingSpeak Cloud	Cycling	80 % of HRmax 18 to 30 min	N = 5 S: both A: n.d.
Guo et al. (2019) [41]	Wristband	Prototype	PPG Sensor	n.d.	Bluetooth	Star	n.d.	Online	Heart rate Blood oxygen saturation	Optimized XGBoost based classification algorithm k-nearest neighbor Decision tree SVM Random Forest Gradient boosting decision tree	n.d.	Running Boys: 1000 m Girls: 800 m	- 3 min. warmup running stage - 2 min. recovery	N = 513 S: both A: 14
Huang et al. (2018) [42]	Calf band	Prototype	Motion Sensor	n.d.	Wireless	P2P	Wearable self-sustained	Offline	Tribo-electrification	Algorithms: SVM Logistic Regression	n.d.	Sitting or Standing, walking, climbing up and downstairs or running	5 sets of: Sitting and standing Walking Climbing up Climbing down Running	N = 3 S: n.d. A: n.d.
Hutnh-The et al. (2020) [43]	n.d.	Prototype	Inertial sensor	AMD CPU 3.7-GHz 16 GB RAM NVIDIA GTX 1080Ti	n.d.	P2P	n.d.	Offline	Human posture and kinematics	CNN SVM	Matlab	Misc.	3 Datasets: Daily and Sport Activities Daily Life Activities Real World	N = 19 S: both A: n.d.
Municio et al. (2019) [44]	Bicycle	Prototype	GNSS (GPS) Speed sensor HR sensor	CC2538: 6TiSCH connectivity nRF52: sensor data collection	6LoWPAN BLE SPI	Mesh network	n.d.	Online	Heart rate Speed	n.d.	n.d.	Cycling	First 17 min. of exercise	N = 17 S: both A: n.d.
Munoz-Organero (2019) [45]	Waist band	Prototype	Tri-axial accelerometer	n.d.	n.d.	n.d.	n.d.	Offline	Human kinematics	Algorithm: CNN	n.d.	Climbing stairs Jumping Lying Standing Sitting Running/jogging Walking	Each exercise performed for 10 min. (except for jumping)	N = 15 S: both A: 31.9 ± 12.4
Qi et al. (2019) [46]	Chest band Wrist band	Commercial	Shimmer3	n.d.	Bluetooth	P2P	n.d.	Offline	Acceleration Heart rate	Two layer recognition First layer: SVM criteria for free and non-free weight activities Second layer: CNN for aerobic and sedentary activities. Hidden Markov Model to provide further classification in free weight activities	n.d.	Aerobic Free weight Posture	Aerobic and posture: - 5 min. each - 3 sets Free weight: - 12 sets	N = 10 S: both A: 30 ± 5
Roslan & Ahmad (2020) [47]	Smartwatch	Prototype	GNSS (GPS) Force sensor Vibrator Motor	MCU n.d.	WiFi	Star	n.d.	Online	Jump Force and speed	OpenHAB	myOpenHAB	High Jump	n.d.	n.d.
Sun et al. (2019) [48]	Smartphone sport belt	Prototype	Accelerometer Gyroscope Depth sensor	x-OSC	WiFi	P2P	n.d.	Online	Human kinematics	Long Short-term memory (LSTM) SVM	Microsoft Kinect SDK 2.0	Baseball: on-field behavior off-field warm up Daily life behavior	n.d.	n.d.
Wang et al. (2018) [49]	Wrist band	Prototype	Tri-axial accelerometer Tri-axial gyroscope Basler acA2000 camera	Smart Bond DA14583 Processor: Cortex M0 Memory 1 MB Flash	BLE WiFi	Star	n.d.	Online	Human kinematics	ML: SVM Principle Component Analysis	Evothings framework Cordova HTTP plugin for Cloud recognition	Racket sports	20 min. warm up 20 smashes 20 short clears 20 long clears	N = 12 S: male A: n.d.
Wang & Gao (2020) [50]	T-shirt	Prototype	ECG sensor Heart rate	Arduino UNO	WiFi	Star	n.d.	Online	Heart rate	Radial-basis function network Probabilistic neural network	n.d.	Volleyball	N/A	N = 100 S: n.d. A: n.d.
Wilkerson et al. (2018) [51]	Chest band	Prototype	n.d.	Smartphone	Wireless	P2P	n.d.	n.d.	Human posture	n.d.	n.d.	Unilateral forefoot squat	Single-leg test	N = 45 S: n.d. A: 20 ± 1.5
Xia et al. (2020) [52]	Wristband	Prototype	MPU6050: Tri-axial accelerometer Tri-axial gyroscope	STM32F103 ARM Cortex M3	BLE	Mesh	n.d.	Online	Human kinematics	Algorithms: ASMV, VSMV, DSMV K-means Clustering Density-Based Spatial Clustering of Applications with Noise (DBSCAN)	Mobile APP LiteOS	Table tennis Badminton Walking	Four type ofTable tennis movements Four types of badminton 20 tests for each movement	N = 5 S: both A: 25 ± 5
Xiao et al. (2020) [53]	Wristband	Prototype	Pulse sensor (photoeletric)	ARM processor	Bluetooth HTTP ZigBee	Star	n.d.	Online	Heart rate	DT algorithm MT algorithm	Android APP Web APP Matlab	n.d.	ECG simulation via Matlab	n.d.
Zhang et al. (2019) [54]	Smartwatch	Commercial	Inertial sensor	n.d.	Bluetooth WiFi	P2P	n.d.	Offline	Human kinematics	Recognition model: K-nearest Neighbor Support Vector Machine Naive Bayes Logistic Regression Decision Tree Random Forest CNN	PyCharm scikitlearn TensorFlow	Table tennis	Table tennis games	N=12 S: both
Zhao et al. (2020) [56]	n.d.	Prototype	GNSS (GPS) Temperature Heart rate	Arduino Mini MCU LinkIt Smart 7688	4G mobile WiFi	Mesh	n.d.	Online	Physiological status	Algorithm: Kalman Filter	MediaTek Cloud	Hiking	Hiking trail 2.1 km long	N = 1 S: male A: 26
Zou et al. (2020) [57]	Smart Glove	Prototype	Inertial sensor	MCU:n.d.	n.d.	P2P	n.d.	Offline	Human kinematics	Recognition model: Dynamic time warping (DTW) FastDTW Half-DTW	Mobile APP	Weight lifting	15 exercises 20 sets each 10 reps per set	N = 8 S: n.d. A: n.d.

AI: Artificial Intelligence; APP: Application; BLE: Bluetooth Low Energy; CNN: Convolutional Neural Networks; DT: Decision Tree; ECG: Electrocardiogram; GNSS: Global Navigation Satellite Systems; GPS: Global Positioning System; k-NN: K-nearest neighbors; MCU: Microcontroller Unit; ML: Machine Learning; P2P: Peer-to-Peer; SPI: Serial Port Interface; SVM: Support Vector Machine; n.d.: not defined.

**Table 4:** Study Results.

Study	Device Type	Application	Processing/ ML Approach	Accuracy	Main Conclusions	Type of Load
Akpa2019 [38]	Glove	Indoor Fitness Activity Tracking	k-NN	Person-dependent: 88% F score: 0.889 Person-independent: 82% F score: 0.830	The device allows one to automatically count the repetition of an exercise, by analyzing the time series of the pressure distribution applied to the hand palm	External
Barricelli2020 [39]	Wristband	Athlete monitoring	SVM-based	SVM-based: n.d.	The Digital Twins applied to SmartFit helped to provide trustable predictions related to twin’s conditions and make easier the optimization of training process.	Digital
Brueck2018 [40]	Wristband	Athlete Hydration Detection	n.d.	n.d.	The real-time sweat rate watch allowed detecting sweat rate with an average error accuracy of 18% compared to manual sweat rate. Future developments using IoT interfaces and physiological sensor may increase the tracking of exercise routines and acute and individualized strategies for hydration.	Internal
Guo2019 [41]	Wristband	Running monitoring	XGBoost-based	97.26% F score = 0.973	The proposed model revealed effectiveness and feasibility compared to previous ones, providing an interesting solution for fitness assessment while running.	External
Huang2018 [42]	Calf band	Physical Activity Recognition	SVM	SVM: more than 80%	The accuracy reach up to 90% for certain activities, while saving 25% of energy in comparison with other sensors. The recognition of human motion was achieved using the proposed approach.	External
Huynh-The2021 [43]	n.d.	Human activity recognition	DeepFusionHAR	DeepFusionHAR: 97.4%	The DeepFusionHAR achieved an accuracy of 97.4% for recognition sport activities. This will help to easily recognize important activities made by humans while exercising.	External
Municio2019 [44]	Bicycle	Cycle-cross training	n.d.	n.d.	The proposed approach help to easily track cycling without the use of 4G coverage, just using an infrastructure-less IoT based platform.	External
Munoz-Organero2019 [45]	Waistband	Human activity recognition	CNN	P-fold cross validation: F score: 0.87	The results presented outperform 8% of those obtained by a p-fold cross validation. The human activity recognition may help future identification of motion and improve the understanding of the quality of movement.	External
Qi2019 [46]	Wristband Chest band	Repetition counting and exercise detection	Neural Networks	Neural Networks: 95.2%	The proposed approach allowed classifying 19 gym activities with a good accuracy. This may help to track exercise and help to design individualized exercises for people, while identifying the load imposed.	External
Roslan2020 [47]	Smartwatch	High jump monitoring	n.d.	n.d.	The validation process was confirmed and the accuracy was improved.	External
Sun2019 [48]	Smartphone attached to belt	On and off-field baseball recognition	LSTM w/ Decision Fusion: SVM w/ Accelerometer:	97.33% 87.33%	The proposed method allowed classifying on- and off-field behaviors of baseball players. This represents a step forward for assessing player’s performance and making decisions to improve the behaviors and design new strategies for each player.	External
Wang2018 [49]	Wristband	Classify at least three different badminton strokes	SVM	97% in stroke recognition 90.3% in clear recognition	The sensors allow capturing motion during playing of badminton, which may help to improve technical skills and individualize the training to fit to each player’s needs.	External
Wang2020 [50]	T-shirt	Hearth rate monitoring	RBFN-LMPN:	73.58%	The monitoring process provided by the solution will help to control the health status of players in real time and detect risk situations early.	Internal
Wilkerson2018 [51]	Upper Torso strap	Injury recognition & prevention	n.d.	n.d.	The model revealed an important accuracy in predicting injury. This is a step forward in injury prevention in sports and in individualizing training strategies to reduce injury exposure.	External
Xia2020 [52]	Wristband	Racquet sports recognition	Multilayer Hybrid Clustering Model:	86%	The wristband allowed detecting racquet movements with good accuracy, which will help to quantify the quality and quantity of movements during training and match scenarios.	External
Xiao2020 [53]	Wristband/Bracelet	Hearth rate monitoring	R-wave recognition:	98.95%	The wearable device will help to detect alert situations early related to health status of players.	Internal
Zhang2019 [54]	Smartwatch	Ping pong movement recognition	Random Forest: k-NN: Decision Tree:	97.8% 95.02% 94%	A great accuracy for recognition of ping-pong movements was found using wearables. This will help to identify the amount and quality of movements and better design training scenarios and manage load.	External
Zhao2020 [56]	n.d.	Physiological status monitoring	Kalman Filter	n.d.	The wristband allowed detecting racquet movements with good accuracy, which will help to quantify the quality and quantity of movements during training and match scenarios.	External
Zou2020 [57]	Glove	Real-time athlete monitoring	n.d.	90.66%	iCoach allows recognizing 15 sets of training programs, and also detecting nonstandard behaviors. This will help to improve the training design	External

CNN: Convolutional Neural Networks; k-NN: K-nearest neighbors; LSTM: Long short-term memory; ML: Machine Learning; RBFN: Radial basis function network; SVM: Support Vector Machine; n.d.: not defined.

## Data Availability

Not applicable.
